# Midwives’ experiences of facilitating normal birth in midwifery-led units in Norway: A qualitative study

**DOI:** 10.18332/ejm/173388

**Published:** 2023-12-11

**Authors:** Liv Remvik-Larsen, Anne Marte W. Gran, Bente Dahl

**Affiliations:** 1 Centre for Women’s, Family and Child Health, Faculty of Health and Social Sciences, University of South-Eastern Norway, Borre, Norway

**Keywords:** midwife, experience, qualitative, normal birth, midwifery-led unit, systematic text condensation

## Abstract

**INTRODUCTION:**

Normal birth has significant benefits for mothers and infants. However, the advancement of technology has led to increased medicalization of childbirth. Midwives play a pivotal role in promoting normal birth, and positive outcomes are seen in births led by a midwife. The purpose of the study is to gain a deeper understanding of midwives’ experiences of facilitating normal birth in midwifery-led units.

**METHODS:**

The study has a qualitative design. It was conducted in 2022 and included semi- structured interviews with seven midwives throughout Norway. They were all currently working, or had previously worked, in a midwifery-led unit. The data material was analyzed using systematic text condensation.

**RESULTS:**

The data analysis resulted in three result categories. The first category concerned the importance of relationships, continuity and a safe atmosphere. The second concerned being a midwife in the hand, in the heart, and in the mind. The third related to having confidence in the physiological process of childbirth, midwifery autonomy, and a common ideology.

**CONCLUSIONS:**

The study highlights several elements that may help to promote normal birth in midwifery units. Relationships, midwifery skills, confidence in normal birth and a supportive collegial environment that fosters midwife autonomy, are prominent factors. These elements were viewed by the midwives as key to their ability to promote normal birth in a midwifery-led unit.

## INTRODUCTION

A normal birth has several positive consequences, both for the mother and the child, such as an improved birth experience for the mother, more rapid initiation of breastfeeding and the forming of a close bond between mother and child^1-3^. The World Health Organization (WHO) defines normal birth as: ‘... spontaneous in onset, low risk at the start of labor and remaining so throughout labor and delivery. The infant is born spontaneously in the vertex position between 37 and 42 completed weeks of pregnancy. After birth mother and baby are in good condition’^4^. However, the use of medical technology in obstetrics has led to over-medicalization and interventions in many healthy women who expect a normal birth^5-7^. Interventions that are performed inappropriately or routinely can, in the worst cases, lead to harm and adverse outcomes for mother and child^5,8^.

According to Norwegian recommendations, prenatal and obstetric care should be continuous and differentiated^9^. The differentiation is to ensure that women’s needs for a varied provision of prenatal and obstetric care are met, and that this is based on a risk assessment according to stipulated criteria and the woman’s wishes^10^. Obstetric care is differentiated into three levels according to the risk of the expectant mother. University hospitals and central hospitals (level 1) provide advanced obstetric, pediatric and anesthetic services, including intensive care for the newborn. Small and medium sized hospitals (level 2) offer obstetric and anesthetic services, and level 3 units comprise free-standing and in-hospital (alongside) midwifery-led units. Level 3 units provide care for low-risk women, levels 1 and 2 provide care for all women, regardless of risk^11^. For the last decades, maternity care in Norway has been characterized by a process of centralization. In 1967, Norwegian women could choose to give birth in 182 different institutions^12^. Today, less than 50 institutions are left. Midwifery-led units have suffered a similar fate. Today, six free-standing units are located within an area of 90–190 km from a hospital and three midwifery-led units are located within hospitals^13^. Obstetric care in Norway is of a high standard in an international context, but improvements are still needed^10^. Preliminary statistics from 2022 show that 30% of births in Norway were induced. Synthetic oxytocin was used to stimulate contractions in 46% of primigravida women and 9% of multigravida women, while 44% of women in labor received an epidural anaesthesia^14^.

Comprehensive antenatal, perinatal, and postpartum care must be in accordance with the WHO’s principles for obstetric care. Normal birth care should be demedicalized and based on appropriate and necessary technology^15^. Midwives are able to facilitate the physiological process of childbirth and limit unnecessary interventions, whilst also providing comprehensive care for the woman in labour^16-18^. A review comparing midwife-led continuity models of care with other care models found that women who received midwife-led models of care were less likely to experience interventions, such as regional analgesia and instrumental vaginal birth, and episiotomy. They were also more likely to experience spontaneous vaginal birth^19^. A study exploring women’s experiences with the promotion of normal birth in a combined low- and high-risk hospital unit described how a positive and supporting approach from the midwife helped them manage labor without pain relief^18^. Thus, midwives play a crucial role in promoting normal birth in all settings.

Nevertheless, midwives sometimes find it challenging to promote normal birth in a high-tech hospital unit^20^. This is partly explained by the lack of evidence-based practice, an overriding focus on procedures, and selection criteria and technology that replace the use of traditional midwifery skills. A systematic review from 2021 shows that a risk-based approach informs practices in obstetric units, and that the loss of midwifery knowledge and skills is one of the barriers to implementing a physiological approach to childbirth^21^.

Previous research has explored midwives experiences with promoting normal birth in a medicalized context. In this study, we explore midwives’ experiences of facilitating normal birth at midwifery-led units in hospitals and free-standing midwifery-led units. None of the units offered caseload midwifery, but some offered continuity-of-care models where women received care from a team of midwives.

## METHODS

The study has a qualitative design, as the purpose is to gain an understanding of the characteristic features and qualities of midwives’ experiences of facilitating normal birth^22^.

### Recruitment and sample

We recruited a purposive sample^23^ by posting information about the study in the Facebook groups *Jordmødre i Norge* (midwives in Norway) and *Hjemmefødsel i Norge* (home birth in Norway). We also had telephone contact with several free-standing midwifery-led units, and after verbally informing them about the study, we emailed information to the contact person in these units.

The inclusion criteria were that the midwives were working or had previously worked in a free-standing midwifery-led unit or a midwifery-led unit in a hospital. Eight midwives expressed their interest in participating, but one subsequently withdrew. Three of the midwives worked in a midwifery-led unit in a hospital, and three worked at free-standing midwifery-led units. One of the midwives worked in a hospital maternity unit, but was included because of her 33 years of experience in a free-standing midwifery-led unit. Six midwives had previous experience from a hospital maternity unit. All the midwives were aged >45 years, and all were currently employed and had 20–40 years’ experience as a midwife. The geographical spread was from Northern Norway to Eastern Norway. In the results section, the midwives are anonymized through the use of fictitious names.

### Data collection

Semi-structured individual interviews were conducted from January to February 2022, and lasted 40–75 minutes. Two authors conducted six interviews, while the last interview was carried out by one of the authors. Five interviews took place remotely, via Zoom, and two were held in person in a public library and at the informant’s home, respectively. An interview guide (Table 1) with a phenomenological perspective on the research interview was drawn up^23^, consisting of four open-ended questions related to the purpose of the study. The participants spoke freely about the main topics, and follow-up questions were asked where necessary.

**Table 1:** Interview guide of midwives’ experiences of facilitating normal birth in midwifery-led units in hospitals and free-standing midwifery-led units in Norway, 2022

**Introductory question** (The aim here was to elicit spontaneous associations, ‘to set the tone’ for the interview)
‘What do you think of when you hear the term ‘normal birth’ – what does it mean to you?
**In-depth questions** (The aim here was to elicit more specific examples in order to explore the concept and experiences)
‘Can you tell us about a situation where you felt that you responsibly promoted and facilitated a normal birth?’ ‘Can you elaborate on how you would handle, for example, a prolonged labor?’
**Direct questions** (The participants were encouraged to reflect on their workplace and the execution of their work)
‘What would you say that working at a midwifery-led unit “gives you” as a midwife?’ ‘What would you say are the main challenges of working at a midwifery-led unit?’

### Analysis

The interviews were recorded using the Diktafon app (an app used to make secure recording on a mobile device) and transcribed verbatim. The transcribed text was analyzed using systematic text condensation (STC), a method for thematic cross-case analysis inspired by Giorgi’s phenomenological analysis method^22^. An example of the analysis process is given in Table 2.

**Table 2:** Example of the analysis process of midwives’ experiences of facilitating normal birth in midwiferyled units in hospitals and free-standing midwifery-led units in Norway, 2022

*Meaning units*	*Code group*	*Subgroups*	*Result category*
‘The most important thing is that the woman feels safe. So, I have to make sure she feels safe. And get to know her a bit in terms of what she wants and needs.’ (Grethe) ‘Simply inviting the couple into a free-standing midwifery-led unit that you can vouch for as being nice and cozy, you can get a lot of brownie points there, with subdued lighting and nice colors on the walls. It’s basically like home. This is a place they want to be.’ (Anne) ‘I think that much of this is down to the fact that we follow all pregnant women in the municipalities during their pregnancy.’ (Marit) ‘And so, we’re present a lot of the time, we have the opportunity to do that. And if we leave the room, we’re nearby.’ (Ingrid)	Relations	Feeling safe and well cared for Environment and surroundings Continuity	Relational interaction; feeling safe, continuity and the environment

STC consists of four steps. In the first step, we read through the transcribed text several times to get an overall impression of preliminary themes, such as preparing for childbirth, time, and hormones. The second step consisted of a systematic review of the text, where we identified meaning units, line-by-line, that helped to elucidate the research aim. These meaning units were sorted into code groups. In the next step, we went through one code group at a time and organized the content of each individual group into different subgroups, all of which highlighted different aspects within the group. The content of each subgroup was rewritten as condensates (artificial quotations), with the intention of summarizing and reproducing the content of the relevant subgroups. In the fourth step of the analysis, the material was reformulated into analytical text and the headings were refined^22^. The analysis led to three result categories. Relevant quotes are included to illustrate the categories. Table 3 gives an overview of the code groups and subgroups established during the analysis.

**Table 3:** Overview of code groups and subgroups of midwives’ experiences of facilitating normal birth in midwifery-led units in hospitals and free-standing midwifery-led units in Norway, 2022

*Code groups*	*Subgroups*
Relational interaction	The importance of feeling safe and well cared for Continuity/being familiar Environment and surroundings; the free-standing midwifery-led unit
Practical and intuitive midwifery skills	Using oneself as a tool Taking care of basic needs/specific measures
Confidence and autonomy	The importance of confidence in birth as a physiological process Autonomy Common ideology/collegial support

### Ethics

The project was carried out in accordance with the ethical principles of the Declaration of Helsinki^24^, and was approved by the Norwegian Centre for Research Data (NSD, ref.: 431412). Prior to the interviews, the participants were sent an information letter. Before the interview, they were informed that participation was voluntary and that they could withdraw from the project at any time without any repercussions. Consent to participate was given in writing or by approving participation in a Zoom meeting by clicking on a link.

## RESULTS

The first category concerned the importance of relationships, continuity and a safe atmosphere. The second concerned being a midwife in the hand, in the heart, and in the mind. The third related to having confidence in the physiological process of childbirth, midwifery autonomy, and a common ideology.

### The importance of relationships, continuity, and a safe atmosphere

The midwives emphasized the importance of forming a good relationship with the women. They argued that if the women felt safe during labor and a closeness was established between the woman and the midwife, this facilitated a positive birth experience. Many emphasized the importance of being present without being a disruptive force, allowing the woman to go into her ‘birthing bubble’. One midwife described how they almost imperceptibly overlapped each other at the change of a shift, without noisy knocking on doors or other disruptive elements. Several midwives spoke of the advantages of the women writing a note or birth plan about what they felt was important during labor, and what they needed to feel safe and well cared for. For the midwife, this could be a shortcut into the relationship:

*‘The first thing I think, which I always have in the back of my mind, is what can I as a midwife do to make this woman feel safe and well cared for? How can I ensure that she can be herself when she is giving birth to her child, and that I can give her, to the greatest extent possible, what she needs and wants? I have to “tune in” to this woman, to the woman in labor.’* (Hilde)

A common feature of the midwives was that they emphasized facilitating continuity. They believed that being familiar with the midwife and with the ward helped to give the women a sense of security, and could increase motivation before and during labor. According to the midwives, this was an important factor in being able to facilitate normal birth. The established relationship also included the knowledge about the individual woman: what she is all about, what resources she has, and where her personal boundaries lie:

*‘If we are lucky, many of the women have had prenatal check-ups with me. We've therefore ensured continuity. So that sense of security and confidence are kind of in the box from the start.’* (Anne)

The environment was highlighted as an important part of the effort to facilitate normal birth. Several participants enthusiastically described in detail the surroundings into which they invite the expectant mothers. Inviting the women into a room where they feel comfortable, with nice colors, subdued lighting, a calm and safe atmosphere, helps to promote normal birth. The midwives also talked about the importance of having enough space to move around and the option to use a bathtub, birthing ball, mats, and a double bed. One midwife had experienced that women who had previously given birth in a traditional hospital maternity ward were more relaxed and more confident in the free-standing midwifery-led unit. Wires and medical equipment were absent, and the midwife had time for the woman and was present:

*‘So just being able to provide a free-standing midwifery-led unit that I can vouch for as being nice and cozy makes me feel that I have done a lot to promote normal birth.’* (Anne)

### Midwife in the hand, in the heart, and in the mind

The midwives described their profession and practical skills with passion. Several referred to being a midwife as an art form, a craft, or a talent. They believed that anyone could learn technical skills and procedures, but that alone is not what makes a good midwife. Some told of an almost intuitive skill that was difficult to put into words, but which was about ‘using oneself’ to read the woman, her partner and the atmosphere in the free-standing midwifery-led unit:

*‘I hear how people breathe, and I hear when their contractions start and stop, and I see how the women move, so that's my most important instrument. I don't have to listen so much to fetal sounds when I know she has good breaks between contractions. Because that tells me that the baby is getting oxygen in between times.’* (Grethe)

All the midwives described a calmness and presence that was in stark contrast to the fast pace of a high-tech hospital maternity ward. Several highlighted the balance between an almost imperceptible presence and jumping into action when required. Experience with normal births in clinical practice over many years had given them confidence to detect any deviations from the normal. Several explained that along with their independent work, there was also a constant awareness of the risk of adverse events. They believed that the ability to think ahead was a prerequisite for providing a high standard of care. The women were transferred to a hospital maternity ward if they expressed a desire for this, or if medical interventions were required:

*‘We always take action if the situation becomes uncertain. If the midwife's gut is telling her that this is not good, then we transfer the woman. But, no, we don't keep them here any longer in the hope of tormenting them into giving birth. And if she loses her courage, and the midwife starts to lose her courage, then it's a vicious circle.’* (Ingrid)

The midwives described how they tuned into the women’s body’s inherent resources and natural hormone production. It is important that the woman is given enough time, that she feels safe and that the body’s reserves can be replenished with fluid and nutrition. Which interventions are initiated and when depends on the stage of the labor. Several midwives compared childbirth to extreme sport, where their basic needs must be met for a good outcome. Everyone highlighted the importance of balancing rest and sleep, activity and changing position. They also highlighted natural pain relief methods, such as acupuncture, massage, warm compresses, running water and baths. All of the midwives emphasized that they take a hands-off approach. No interventions, such as catheterization, vaginal examinations or amniotomy, were initiated without a clear indication:

*‘But the changing of position, especially at the dilation stage, trying different positions, standing, kneeling, some want to rest, on a bean bag, lying on your side, all of that ... well, birth is movement. And it's important, and it’s important to move.’* (Marit)

### Confidence in the physiological process of childbirth, midwifery autonomy, and a common ideology

All the midwives had a strong belief in the physiological process of childbirth. Some emphasized that if the midwife does not have this belief, this is transferred to the woman. Preparing the women well for childbirth was emphasized by several midwives. This entailed allaying any fears about the birth process, and reassuring the women and their partners that giving birth is a natural process that the female body can handle. One of the midwives referred to the ability to make women understand and believe that they can do it themselves as an art. Some described the efforts they made to maintain the women’s belief in their own bodies and to listen to their instincts. They knew when to praise and encourage the women:

*‘It’s an art, I think, to help the woman see that she can do it herself, and to believe in it. ... “When you think you're exhausted, you're halfway there. Then your body can at least do twice as much. And you don't believe it, but I do.” Then you see that they get on board in a way.’* (Bente)

The midwives said that they feel that their profession and their identity as a midwife are to some extent threatened by invasive procedures and the introduction of technology to the free-standing midwifery-led unit. They described feeling reassured by the knowledge that their colleagues share a common ideology, and they discuss midwifery, research and philosophy. One of the midwives emphasized the importance of knowing that at shift change, the next midwife takes over responsibility for the woman in labor in the same spirit and in the belief they can facilitate normal birth. The midwives were very aware of the conditions for using a partograph, for example not starting it too early. They said that this is one of several factors that help to optimize the potential for the woman giving birth with as few disruptions as possible:

*‘You tend to cheat a bit on the partograph, yeah. And you have to do that because otherwise hardly anyone would be able to give birth in peace. And don't open the partograph too early. You can of course be lucky if you do that, but it's a bit sad that it has to be like that.’* (Nina)

The midwives emphasized the importance of maintaining a high standard of care. They considered themselves a vulnerable group, and it was important to them that they could not be accused of anything. This also applied to protecting the women’s right to co-determination. The women were encouraged to take their share of the responsibility for their own birth process, but it is the midwife’s job to reassure her that her body can give birth to a child. If the decision is made that a woman will give birth in a free-standing midwifery-led unit, a normal labor is expected. Most of the midwives had experienced challenges in facilitating normal birth when they were previously employed in a hospital maternity ward. Several midwives mentioned an overriding risk surveillance approach and fear that the unborn child is in danger, and that this insecurity transfers to the women. Some said they did not speak the same language as the midwives in the hospital maternity wards, or that they felt misunderstood by them. At worst, they felt that they were polar opposites. Reassurance is important for maintaining progress in childbirth, but the views on the place of technology in this process varied. One of the midwives had recently conducted her annual practice at a hospital maternity ward and shared the following reflection:

*‘We practice in the maternity ward once a year, you know, it's in our criteria. And [during the hospital stay] I always think that it will be good to come home to reassurance. … We're not there to manage a woman's labor, we have time, not least, time is extremely important. Give the women time and reassurance. It's incredibly important.’* (Marit)

## DISCUSSION

A reassuring relationship was a key element of the midwives’ efforts to facilitate normal birth. The importance of good relationships in obstetric care has previously been highlighted. Hunter et al.^25^ point out that in addition to other conditions that contribute to promoting normal birth, the importance of the caring relationship in particular must be recognized and considered. In order to form a reassuring relationship, the midwives placed an emphasis on providing continuous antenatal, perinatal, and postnatal care. Continuous care can increase the likelihood of a spontaneous vaginal delivery and reduce the number of women receiving epidurals, episiotomies, and instrumental deliveries^26^.

Comprehensive obstetric care is one of the goals of Norwegian health policy^9^, but this is sometimes difficult to achieve as the responsibility for this care is divided between the primary health service and regional health authorities^10^. However, many pregnant women are followed up by one midwife, or just a few, during pregnancy, which provides a continuity. Through prenatal care, the midwives we interviewed gained knowledge about the individual woman and her resources, in parallel with childbirth preparation work and motivation. Thus, the relationship was already established when labor started, which can make it easier for the woman to enter her ‘birthing bubble’. Vedeler et al.^27^ indicate that women want to feel a sense of security during labor, without unnecessary stress and interventions. The midwives in the free-standing midwifery-led units had time to be present during the women’s birth. Their continuous presence during the birth gave them more opportunity to support the woman and her partner, and to make observations. This presence can help to facilitate normal birth and shorten labor. It can also reduce the number of cesarean sections, instrumental deliveries and the use of epidurals, giving a better birth experience for the mother^28,29^.

The environment was highlighted as playing an important role in the reassuring relationship. The importance of the environment is also examined in previous studies^30,31^. The midwives talked about how women who had previously given birth in a hospital maternity ward were more relaxed when they arrived at the free-standing midwifery-led unit because the absence of medical equipment gave them a greater sense of calm. However, experiencing a ‘sense of security’ involves more than ‘being safe’. For some, the sense of security entails having technology close-by, while for others it is the absence of technology^27^.

The midwives enthusiastically described how being a midwife is about more than handling practical skills. It was difficult to put into words how they work, and terms such as ‘the art of midwifery’, ‘using one’s whole self’, ‘tune in’ and ‘midwifery talent’, were used. The midwifery profession represents an old craft and traditions, which are sometimes felt to be under threat from the gradual introduction of technology, including in low-risk units. Physical proximity to this equipment can influence the threshold for putting it into use. Advanced medical equipment is vital for some, but it can also lead to unnecessary interventions or continuous monitoring, which in turn can lead to adverse outcomes in healthy pregnant women^32^. The absence of medical technology challenges the midwives to use their own senses as tools, and their continuous presence gives them a sense of security, where time is an important resource. This does not entail excluding technology, but recognizing when it needs to be used. This is in line with the WHO’s evidence-based recommendations for intrapartum care^6^.

The midwives in this study are aware that they occasionally use strategies that stretch the limits for certain procedures, without this increasing the risk for the women. Making conscious choices during childbirth can reduce unnecessary interventions, thereby helping to optimize the potential for a normal birth^19,33^. One such example is conscious use of the partograph. It is not necessarily expected that labor will take place within a linear timeframe as suggested by this graphical record of key data. A normal birth often takes time. If mother and child are doing well, calmness and patience are a resource. If the partograph is started too early, it can limit the potential for giving birth without disruptions. There is little evidence of the independent significance of the partograph for the outcome of a birth^34^. Several studies confirm that midwives who take a physiological approach in a risk-managed ward tailor their choice of action in a similar way^20,21^. Allowing laboring women enough time and calm to give birth with as few disruptions as possible, was highlighted. Here, a dilemma arises between being preemptive, identifying acute situations and acting accordingly, whilst also holding back. Some highlight the desire to ‘be a midwife’. This implies a need to practice one’s profession, without the level of tension and tight time frames found in emergency nursing or obstetrics.

Midwives’ confidence in birth as a physiological process is crucial for promoting normal birth^16,19,25^, and it also plays a role in their choice of workplace. Working in a free-standing midwifery-led unit required not only a belief in the women being able to give birth, but also in themselves and their own knowledge. According to Hunter^25^, confidence in birth as a physiological process requires a safe and reliable relationship between the members of the midwifery team. The midwives we interviewed highlighted a common ideology as an important reason for facilitating normal birth. The security of knowing that colleagues shared the same approach to birth and the opportunity for professional discussion was highly valued, and a strong emphasis was placed on the art of transferring this to the women. Both WHO^4^ and the International Confederation of Midwives (ICM)^35^ emphasize that midwives must work to promote normal birth, which the midwives do by providing continuous antenatal care for the women. This gave the midwives the opportunity to provide information and to prepare and motivate the women for a normal birth, and they thoroughly enjoyed watching the women’s progress throughout their pregnancy.

Shared collegial beliefs and positive cooperation can be at odds with previous research where midwives working in maternity wards find that impatient colleagues ruin the elements of time and patience, which are needed in a normal birth^20^. Several had experienced contradictions between midwifery theory and practice in previous jobs at a hospital maternity ward. They talked about the experience of being caught between technological procedures and professional autonomy. They perceive it as frightening when confidence in technology comes at the expense of clinical insight and knowledge acquired through many years of practice. The ward’s framework can govern how the midwives can facilitate normal birth. The reasons for the midwives’ previous experiences of resistance to a physiological approach to childbirth are complex, but include rigid timeframes and lack of continuity^36^. Centralization at organizational level also plays a role, as does the focus on risk surveillance behavior within wards at a professional level^21^. An overriding risk surveillance approach in hospital maternity wards is a factor that is known to inhibit facilitating normal birth in hospitals^20^. At the individual level, lack of knowledge, lack of autonomy and the feeling of ownership of one’s own birth process are barriers to the physiological birth process^21^. Conversely, women who give birth in a midwifery-led unit have made a conscious choice about their own childbirth experience.

### Strengths and limitations

The use of a qualitative method with thematic analysis was a suitable approach for this study as we aimed to gain knowledge on the experiences of a small group of midwives in Norway^22^. All of the midwives had positive experiences in promoting normal birth. They had extensive experience from a midwifery-led unit and were enthusiastic about their profession. This allowed for a rich dataset to be produced despite the small purposive sample. Nevertheless, a broader sample including midwives from non-Western cultures as well as less experienced midwives probably would have strengthened the study. Further, we used Zoom to conduct several interviews. This probably made it challenging to interpret non-verbal language, but on the other side, it enabled us to include midwives from a wide geographical spread. One of the study’s strengths is the close collaboration between the authors throughout the process. This helped prevent the authors’ own opinions and preconceptions becoming prominent.

## CONCLUSIONS

This study sheds light on several aspects that help to facilitate normal birth in midwifery-led units in hospitals and free-standing midwifery-led units. Reassuring relationships, midwifery skills and the importance of believing in the birth process in a supportive working environment that facilitates the midwife’s autonomy, are prominent factors. The midwives had positive experiences in promoting normal birth. However, they also highlighted the challenges of working in a similar way in medicalized contexts.

## Data Availability

The data supporting this research cannot be made available for privacy or other reasons.
